# The experience of parents of children with rare diseases when communicating with healthcare professionals: towards an integrative theory of trust

**DOI:** 10.1186/s13023-019-1134-1

**Published:** 2019-06-28

**Authors:** Beni Gómez-Zúñiga, Rafael Pulido Moyano, Modesta Pousada Fernández, Alicia García Oliva, Manuel Armayones Ruiz

**Affiliations:** 1 Estudis de Psicologia i Ciències de l’Educació, Universitat Oberta de Catalunya, Rambla Poblenou, 156 08018 Barcelona, Spain; 2 Edificio Departamental de Humanidades y Ciencias de la Educación I (Edif. A). Planta 2, despacho 14, Universidad de Almería, Ctra. Sacramento s/n, La Cañada de San Urbano, 04120 Almería, Spain; 3 PSiNET Research Group, Universitat Oberta de Catalunya, Barcelona, Spain; 4 eHealth Center, Estudis de Psicologia i Ciències de l’Educació, Universitat Oberta de Catalunya, Ctra. Sacramento s/n, La Cañada de San Urbano, 04120 Barcelona, Spain

**Keywords:** Doctor-family communication, Rare diseases, Qualitative research

## Abstract

**Background:**

Given the inherent complexity of rare paediatric diseases and the sensitive emotional context of the situations they create (due to the patients’ age and the tense uncertainty surrounding the progression of the disease), communication between the adults involved is a key tool in the efforts to provide these children and youths a better quality of life. We conducted ten interviews with families of children with rare diseases, in the aim of exploring how communication between doctors and patients affect their daily lives.

All participants, members of FEDER (a Spanish federation of associations of patients with rare diseases) were invited by phone or email to participate in a semi-structured interview including questions on clinical information, communication experiences with healthcare professionals, and the impact these had on the interviewees’ relationships with them. To analyse these interviews, we used the ‘grounded theory’ methodology and open and axial text coding techniques, in addition to those identifying the properties and dimensions of the categories formulated.

**Results:**

The core category we have proposed is ‘adjustment of mutual trust’, with said category describing the attitude and behaviour of doctors who inspire trust in the parents of paediatric patients diagnosed with a rare disease. More specifically, said behaviours or sources of trust are: appearing human, sensitive and empathetic; showing transparency and communicative openness; being supportive of parental proactivity; and being available to families at all times.

**Conclusions:**

Trust is the cornerstone of parent-doctor communication in the field of children with rare diseases. If the sources of trust are present, they create a degree of trust that bolsters both parties in the search for a common goal: providing the child with the best possible care.

**Electronic supplementary material:**

The online version of this article (10.1186/s13023-019-1134-1) contains supplementary material, which is available to authorized users.

## Background

In Europe, a disease or disorder is defined as rare when it affects less than 1 in 2000. The majority of rare diseases appear at paediatric age and frequently entail different degrees of disability. When dealing with rare diseases affecting children and youths, the main concern, the core mission shared by parents and healthcare professionals alike, is to pursue the greatest quality of life for those suffering from said diseases. For many parents, caring for a child with a rare disease entails lifelong challenges and personal sacrifice, often with scant official support, limited access to health services and a complete absence or relative lack of experienced professionals able to provide the required care and take the right decisions [1–3].

Given the inherent complexity of these diseases—their “rarity”—and the sensitive emotional burden inherent in the situations they create (due to the patients’ age and the tense uncertainty surrounding the progression of the disease), communication between the adults involved becomes a key tool in the efforts to provide these children and youths with a better quality of life.

Although there is little published research tackling the issue of communications between the doctors and family members of patients in the specific case of rare diseases, we can leverage the abundant literature on doctor-family communications in the field of paediatrics, mainly in cases of patients with chronic illnesses, to find points of connection to provide the framework for our contribution. Rare diseases and chronic diseases have in common the origin or cause of the disease, its symptoms, duration, incidence in the patient’s quality of life, and possibility of cure or stabilisation of the disease. In this sense, rare and chronic diseases share a number of common burdens such as illness experience, biomedical facts, illness perception, likelihood of cure, etc. Thus, the conditions under which these occur may also be similar, and coping strategies for meeting with health professionals and the relationship between health information and health communication are crucial in this context.

Almost thirty years ago, Richard Street’s [4] research into communication between parents and paediatricians noted that certain aspects of communicative behaviour had a greater impact upon parental satisfaction than others. The author outlined a model comprising three communication elements: (1) *informativeness*: the quality and quantity of the medical and healthcare information provided; (2) *interpersonal sensitivity*: behaviours in the affective domain that reflect concern for the feelings of parents and children; and (3) *partnership building*: the degree to which parents participate, give their opinions and make suggestions. Street [4] pointed out that parents’ perception of these three elements determines their satisfaction with the medical care their children are receiving.

Years later, Galil et al. [5] took a more in-depth look at doctors’ skills in displaying a feeling of real concern for their paediatric patients and their parents, which is what provides the basis for communication and cooperation with them. These affective displays become even more important in rehabilitation processes, when parents and doctors must cooperate intensely and over a prolonged period of time, because these affective displays break down any formal distance that may exist between them and enable an atmosphere of rapport and close interpersonal relationships, argue the authors.

Furthermore, displays of affection and real concern by physicians boosts parental confidence and trust in both rehabilitation processes and their own ability to successfully conduct them. One of these authors’ working conclusions is of great importance to us: the feeling that doctors are truly concerned and show emotional closeness is something that empowers parents.

A number of studies have pointed to the ideal features of a paediatrician’s communicative style, including active listening aimed at children and their parents, direct and honest speech, and spending the time necessary to explain every detail and offer more comprehensive information [6–10]. Similarly, they have highlighted negative aspects, such as giving ‘bad news’ abruptly and insensitively and concealing information [6, 11].

A study in Australia [12] of 30 families with children diagnosed with genetic metabolic disorders indicates that families’ emotional and economic stress and the perceived need for greater social and psychological support were accompanied by greater proactivity, manifested in the parents’ wish to maintain better communication and coordination with healthcare professionals and to have access to more adequate information.

Parents of children with rare diseases seem to display more accentuated proactivity than other parents. These parents want to feel they are members of the team overseeing their children’s medical care and that their needs are being met at all times. Several papers have show that parents’ feelings of frustration and their concern about professionals’ lack of understanding of the disease have a negative impact on the quality of and access to care [3, 13–16]. This is why parents often feel that they have no choice but to assume the role of ‘experts’ in all aspects of their children’s health [16–18].

Additionally, a number of studies [19, 20] have indicated that mutual support between families in similar situations with regard to their children’s rare diseases, the encouragement they given one another, is of crucial importance. It provides parents with a kind of shared social identity, a feeling of belonging to a group, that enables them to deal with the situation better, alleviates their stress and makes them feel more empowered to manage their children’s’ needs.

Parents’ proactive behaviour was identified by Dalby [21], who indicated that those with a family history of rare diseases were more open to genetic testing than those of healthy children. Receiving the results of these tests to put an end to their diagnostic odyssey comes as a great relief to them [22]. These are parents who wish to become actively involved in research into their children’s diseases [23], to possess all possible available information and decide what, when had how to inform their children. In this regard, parents are regarded as logical information filters for their children [24], as they always want the best for them and wish to take the best decisions on the basis of the information they receive from professionals and from the children themselves, when the latter’s age so permits [24–26].

Based on the above literature review, this study aims to explore doctor-family communication in the case of children with rare diseases. To do so, our basis will be the direct experience of a set of parents, and we analyse what characterises doctor-family communication within the context of caring for these children. Due to the lack of prior research into the matter, our work has an exploratory approach, so as to: 1) define the key elements upon which parents base their communicative relations with the doctor and which determine whether these relations are or are not satisfactory; and 2) propose a model that integrates them in an understandable way and renders a structured account of the communicative dynamics between the parties.

## Method

### Study design

This paper shows the results of a qualitative data analysis performed on a corpus of text transcriptions of ten individual interviews. To carry out the analysis, we have used some procedures typical of the methodology known as ‘grounded theory’, which is used in studies on issues closely associated to the focus of our own [27]. Specifically, open and axial text coding techniques have been employed, as has identification of the properties and dimensions of the categories formulated [28] (Strauss and Corbin 2008).

### Participants

The participants were ten parents of children with rare diseases. Given that paediatric doctor-patient communications take place mainly between the doctor and the children’s parents, we decided to choose mothers and fathers as our participants.

From amongst the potential families, we took into account whether they were parents who, due to their involvement with their children’s diseases, were available and interested in participating in the study, and excluded those parents who, for reasons of geographic location or the state of their children’s illnesses, could not commit to the interview or the time that it would take up.

We recruited participants through the Spanish Rare Diseases Federation (*Federación Española de Enfermedades Raras*, FEDER), more specifically with the help of one of the Federation’s psychologists. The criteria for inclusion were that participants (mothers or fathers) regularly attended their children’s check-ups or programmed medical appointments, and that the diseases of participants’ children were varied enough to cover the greatest possible number of involvements and peculiarities that such diseases could present. We did not take into consideration any particular age range for the children, because it was not this variable that was important: the important variables were those associated with the communication process between doctors and families.Participants were young middle and upper-middle class parents, aged 30 to 40. Six couples were married, two were divorced and two were unmarried. The level of studies completed ranged from secondary to higher education, and they lived in Barcelona and the surrounding area. Eight of the ten parents interviewed had created an association which was the first for their son or daughter’s disease.

The psychologist contacted the families and explained the study’s research goals and focus. Subsequently, if the families agreed to participate, the researchers sent them a written summary of the project. Eighteen families expressed an interest in taking part and, in the end, interviews were arranged with ten families—more specifically, eight mothers and two fathers.

Participants provided written informed consent to have a research team member interview them, and received €70 as compensation for their involvement.

### Data collection

To prepare for the interviews, basic information on the child’s disease was collected in advance. A. G. O. carried out the interviews over December 2016 and January 2017 at the FEDER headquarters, except for two families, for whom the location was changed to fit in with their availability.

Interviews commenced with information on their duration, signing of the informed consent, and permission to record them. They continued with some general questions on the child, to then move on to questions on doctor-family relations and communications. They were semi-structured, with a guide previously drawn up by the researchers (Additional file 1), and participants were asked to express themselves freely. Interview questions included: “Has the type of your relationship with the doctor been helpful with the treatment and the day-to-day realities or other aspects of the illness?”, “Do you really understand what the doctor tells or explains to you?”, and “Are the medical reports understandable for you?”. One single interview, lasting between 60 and 90 minutes, was held with each participant.

### Data analysis

The first stage of analysis consisted of carefully reading through the ten interviews and initial, line-by-line coding of the entire corpus. The codes act as labels that we researchers assign to those data fragments (in our case, words, sentences and paragraphs from the transcriptions) that, for some reason or another, attract our attention. In this study, more than 600 codes were assigned at this stage.

Upon conclusion of the initial coding of all the material, the codes were reviewed and brief discussion notes formulated on those we regarded as most significant, although there were a number for which these notes had been made before completion of the coding of the text corpus. More specifically, notes on 76 codes were drawn up. At the beginning, due to their newness, the first interviews were full of codes, but then there was a gradual appreciation of how many things were repeated, although new ones did appear in every interview.

These thoughts—known as ‘memos’ in grounded theory argot—are the basis for grouping the codes together on the basis of some kind of affinity, according to a range of different criteria (cause and effect relationships, temporal sequences, part-whole relationships, etc.). Some codes appeared strongly above others, subsuming many more, and other initial codes were simply discarded because we could not appreciate any clear link with the more powerful codes. As a code gained strength and appeared as an ‘umbrella’ encompassing others, this code became a ‘category’. This was the second stage of analysis.

Stage three saw us taking a more in-depth look at each category, one by one, and reviewing the memos drawn up on the initial codes grouped into each category. This was done to make sure that they were properly classified and to find the link between the category as a whole and the other categories, as they must be explicitly interrelated by means of connecting statements. The goal here was to find a core category that would act as an axis to articulate the other categories and as nucleus around which the theory to be constructed turns.

A category achieves ‘data saturation’ when researchers have managed to specify all its significant ‘properties’ and the ‘dimensions’ of said properties. This should be attempted for all the study’s important categories, and is imperative in the case of the core one. This explains why grounded theory requires that the data be produced gradually, as the theorisation (the conceptualisation/categorisation) of the phenomenon progresses. The idea is to seek new data that permits the refining of concepts/categories when their properties and dimensions are revealed. And, in this search for new data, the sampling criteria are based on the suggestions or ‘suspicions’ arising from the theorisation already under way: this is why it is known as ‘theoretical sampling’.

## Results

Based on the aforementioned analysis, 21 categories were built and grouped, in turn, into five blocks, as listed below:

- ***Block A ‘Families’.***
- ***Block B ‘Doctors’.***
- ***Block C ‘Families and information on their child’s disease’.***
- ***Block D ‘Parent-doctor communication’.***
- ***Block E ‘Associations’***

See Additional file 2 for a detailed list of the 21 categories and subcategories.

**Fig. 1:**
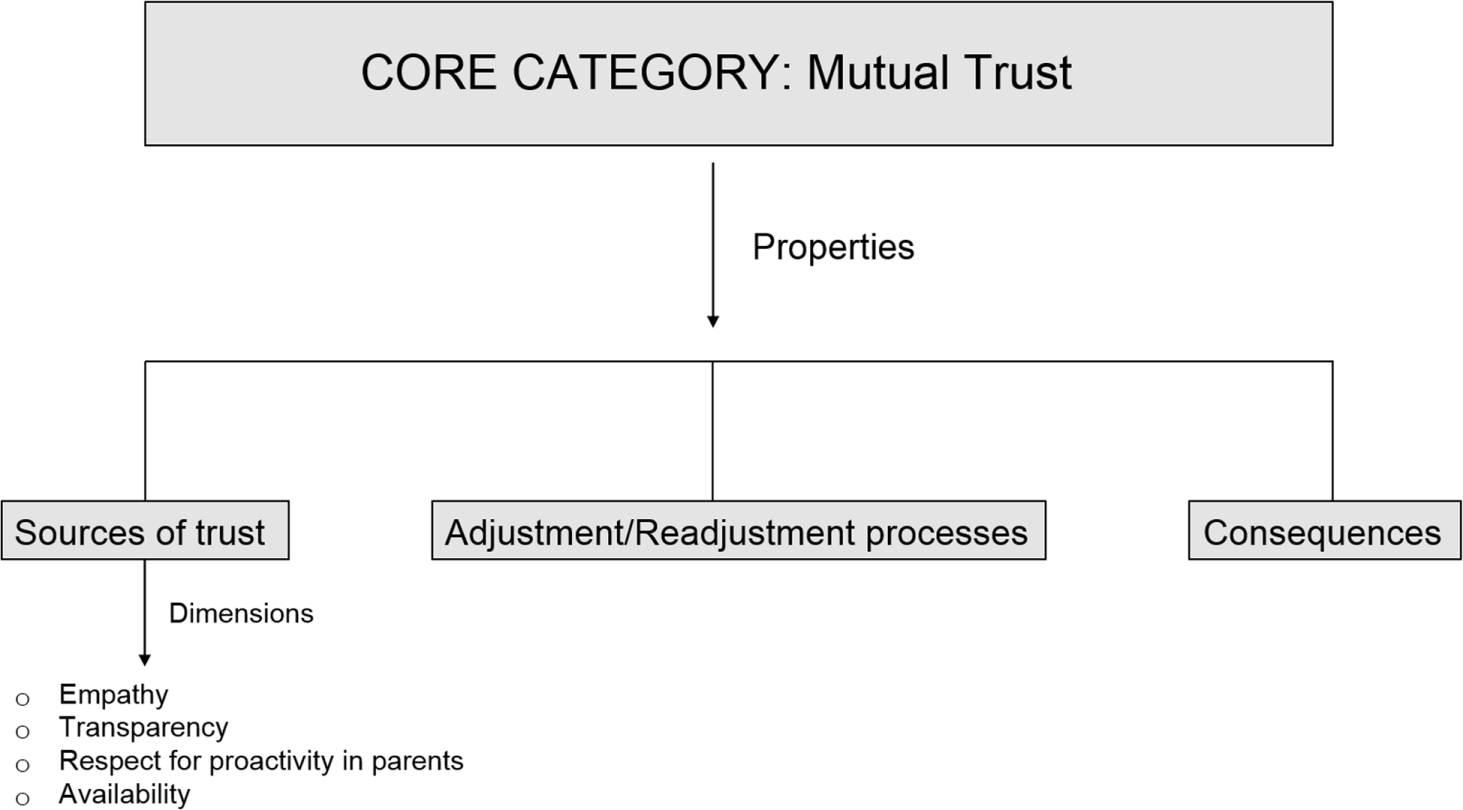
The four dimensions of the sources of trust

The core category proposed here is ‘adjustment of mutual trust’, a two-way process: a) parents’ trust in doctors, and b) doctors’ trust in parents. In this article, we shall be looking at the former: ‘parents’ trust in doctors’.

Hsiao, Evan and Keltzer [29] singled out trust as a key factor in facilitating communication and provided examples of how it can be harmed when, for example, doctors fail to acknowledge an error, something that had already been pointed to in an earlier study [11]. Communication is fluid, rich and effective to the extent that it is based on mutual trust.

For us, this is the core category of our study because, in addition to it being the one with the most (and most intense) relationships with the other categories constructed in the data analysis, it is the category that we can best categorise, given the data available, identifying its ‘properties’ and the ‘dimensions’ of these properties (Fig. 1).

The core category of our study has not yet reached the point of ‘data saturation’. We believe that there is still a shortfall of data keeping us from being able to present a complete categorisation of all its properties and dimensions. However, in at least one of these properties, which we call ‘sources’, we *have* achieved saturation: in other words, we have enough data to allow us to present this property in sufficient detail.

Set out below are the results of the ‘sources’ property (trust is always based on *something*), although our analysis allows us to point to some other properties of the category, such as ‘adjustment/readjustment processes’ (the degree of trust may vary based on the evolution of the parent/doctor relationship) and ‘consequences’ (an increase or decrease in trust may, in turn, cause changes in other aspects of the relationship or even in the self-perception of each of the parties).

Ten interviews were enough to identify the attitudes and behaviours of doctors that parents take as ‘sources’ upon which to base their trust in the former. We do not believe that more interviews would point to other sources: at most, they could provide more detail on those already identified. According to our theoretical characterisation of the ‘sources’ property, its entire variability occurs in four dimensions, as we shall explain below, which describe doctors’ attitudes and behaviours that inspire trust in parents of paediatric patients diagnosed with a rare disease.

### Trusting those appearing human, sensitive and empathetic

A number of qualitative studies on the way in which healthcare professionals communicate with their paediatric patients and the latter’s families [29–31] agree in pointing to three ideal characteristics of said communication: showing respect and compassion, providing emotional support, and boosting their knowledge of patients and parents as individual human beings. Our study confirms this. Parents have greater trust in doctors when they perceive that they are treated with sensitivity, tact and as human beings:

*I … I did ask once … in a talk I asked, umm … how much training time is given to doctors to train them in being people, in humaneness?*

Parents have more trust when they are convinced that the professional in question knows how they feel regarding their children’s illness and that he or she is truly interested in aspects that go beyond purely clinical ones with regard to their children and to themselves as parents. They need to feel they are being listened to:

*So the doctor’s going … (pretending to type). Just writing and writing. So, like, five or ten minutes go by and, of course, the family’s explaining. And then I said, “Hey, have you taken on board anything I’ve said? You haven’t even looked at me!”. It was all really difficult.*

Parents need to feel that professionals are aware of the affective impact of the disease upon them and show it through the way they talk to them:

*And everything in the way they convey things, when they say … a bit of empathy! It’s about having empathy when you say, “Look, he’s probably got a very rare disease. Don’t worry, here in Barcelona, at Hospital X and Hospital Y they’re treating about 100 kids ( … )”. That’s the way to convey it to the family. What you can’t say is that they might not make it and that … it’s the way you say things.*

Parents will have trust in the doctor to the extent that they perceive the latter’s capacity for empathy, tact and sensitivity. Some noted that it sometimes seemed that the doctor forgot that he or she was treating a child or that whatever happened during treatment would have great emotional repercussions for the patient and the family. So, trust depends on the degree to which professionals show that they are aware of the extent of parents’ suffering, because the parents’ experience is clearly one of suffering, suffering caused by four feelings: physical and psychological wear and tear and exhaustion; abandonment and loneliness; distress (due to lack of knowledge and uncertainty); and a sense of guilt.

Parents suffer because, firstly, they reach the end of their tether. Their children’s illness requires so much attention, time and energy that the physical and emotional wear and tear will sooner or later take its toll. To alleviate the burden of this feeling of “I can’t take it anymore!” and to lessen their suffering, they find support, where possible, in family members, to whom they delegate some day-to-day chores to allow them to rest awhile. This emotional burden and the everyday difficulties in caring for the child are described in detail by Hentinen and Kyngäs [32] and by Trulsson and Klingberg [33].

Secondly, another obvious source of suffering is the feeling of being abandoned to their fate, that they are experiencing their child’s illness on their own, that nobody understands them and no-one can help. Somanadhan and Larkin [34] quoted parents describing this experience as “feeling like you’re in no man’s land” and “the feeling that the future is unknown”.

To alleviate the burden of this feeling and to combat the suffering it causes, parents contact associations or, depending upon the case, found a small one themselves. Within this association, and also outside of it, they can share experiences and exchange knowledge with other parents in the same situation, which helps them see and feel that they are not alone, that there is someone there by their side, and that they have not been abandoned. Parents also seek this companionship in the doctor in charge of their children’s case, although they will not always find it:

*If the doctor tells them, “There are clinical trials. There’s a chance … ”, then he’s your best friend. But if he says, “There’s nothing; we can’t do anything. There’s absolutely no way forward. Research is progressing very slowly … ”. All of that: you’re playing a part in the fact there’s no research, that everything’s going slowly, that your child has little life expectancy. Like, how do you get that into your head and take it on board? So that kind of doctor’s not a good friend. It’s not that he’s a good or bad doctor, but … he’s necessary … . So, for professionals, I think what’s important is, firstly, the language: use clear and calm language with the family because the family needs that peace of mind, that companionship …*

Thirdly, parents suffer because they are distressed that they do not know everything they think they should know about the disease and because they do not know what will happen in the future, how the disease will progress, or what will happen when their child reaches adulthood. This uncertainty sometimes leads to almost obsessive behaviour in the search for information. They are convinced that the more they know, the better they can help their child, and they sometimes attain almost the same level of technical and/or clinical knowledge as the professionals. With regard to the disease, they find it difficult to accept the fact that there is much that scientific research is still unable to explain, and they also suffer because future prospects are out of their control.

Uncertainty, anxiety and the fielding of loneliness are accentuated during the

‘diagnostic odyssey’ [35] (Dudding-Byth 2015: 624). To combat this third feeling, this anxiety of uncertainty that makes them suffer so much, some put themselves in the hands of the doctor, trusting that he or she will resolve all the doubts that can be resolved, although they sometimes come across the problem of finding the information hard to understand.

Lastly, parents suffer because they harbour, to a greater or lesser extent, some kind of feeling of guilt because of what has happened to their child. This was explicitly acknowledged by only one interviewee and almost went unnoticed in the initial analysis. In the subsequent review of the codes, however, it became the subject of careful consideration. It is clear that there is no reason for any parent to feel that they are guilty of their child having one of these rare diseases, but knowing that a disease is due to genetic factors may undoubtedly lead to such a sense of ‘guilt’ in a parent and, as unjustifiable and absurd as it may seem to us to harbour this feeling, the fact is that it exists amongst some parents. A mother’s or father’s feeling of guilt about what is happening to their son or daughter could help explain the behaviour that such parents may display with regard to the search for information or to their complete dedication to their child—at the cost of their own health. In any case, although this latter sense of guilt would appear to us to be of great importance (and we believe that further research into it is called for) such ideas are still no more than mere hypotheses, as there is scant explicit trace of it in the managed data. However, this does not mean that it is unimportant: quite the contrary, perhaps.

In summary, parents have greater trust in a doctor when they believe he or she is really interested in aspects that go beyond purely clinical ones with regard to their children and to themselves as parents. October et al. [36] saw clear evidence, in their study of the presence of parents at professional medical congresses and workshops, that parents want to feel understood and have their fears and concerns addressed.

Parents need doctors to be aware of the obligation, the need or the irrevocable decision they have taken to do everything possible for their children, even if this implies sometimes superhuman efforts or involves putting their own health, financial security, or whatever else, at risk. They will have more trust in a doctor capable of putting themselves in the difficult position they find themselves in. Following the issuing of the diagnosis, a whole raft of changes occurs in the life of the affected family: in work, economic impact, the securing of support from members of the family, the risk of a breakup in the couple, etc. Everything is turned upside down, everything has to be rearranged. Decisions are taken on an immediate-term basis, on what is most urgent, but also with a view to the longer term. It may be these transformations that, given their depth and urgency, give rise to the need to benefit from the support of those who have experienced, or are still experiencing, the same thing.

### Trusting those displaying transparency and communicative openness

Generally speaking, it can be said that information is the key capital forming the transactional basis of meetings between parents and doctors. The other ‘capital’ would be the affective tone the parties take with one another, the degree of mutual trust being the result of these transactions in each encounter. Here, it is perhaps truer than ever that information is power. In the hands of the doctor, it allows him or her to have an influence over the parents to ensure they behave in the way he or she deems fit. In the hands of the parents, on the other hand, information empowers them and allows them to curb the doctor’s power.

Metcalfe [37] and Metcalfe, Plumridge and Coad [38] state that most parents prefer to have accessible information that is free of technical or scientific jargon. Likewise, they underline the need to be informed as the basis on which parent-doctor relations are built. A number of studies have made it clear how parents of children with rare diseases often search the Internet, initially, to find information on the disease and the available resources [39, 40]. The active, ceaseless search for information is a characteristic of proactive/empowered parents who, as we shall note later on (see Section 3.3.), place more trust in those doctors who respect this proactivity.

Other studies [41, 42] point to how this sometimes obsessive search for information can be explained by certain experiences the parents have had with the healthcare system or by the perceived incompetence or indifference of medical staff, which would lead them to feel forced to make themselves the ‘experts’.

Parents trust the doctor to the extent that they are convinced that the latter is telling them everything he or she knows about the disease and their child’s treatment and is not hiding information. Glenn [43] described the case of a number of parents who felt frustration in this regard. One mother confessed to this researcher that “the worst feeling is when I have been managed by a doctor or a nurse; where they decide that they are going to a give me a limited amount of information instead of all the information” (id., p.21). However, some parents appear resigned to the fact:

*[we receive] very little information from the doctor, very little. ( … ) The doctor takes the decisions. We can’t … we can’t decide because we don’t have enough information. I mean, if you don’t have information, you can’t decide.*

Other parents, doubtless those with a highly proactive profile, say something very different:

*I need to know everything and more, much more, and, and … and I love it when a doctor explains things to me and explains them well. I love it! When a doctor explains something well, when you understand and enjoy it, even if it’s something negative about your kid, well, it gives you a clearer idea of what’s happening to your child.*

Within this dimension of transparency and informative openness, we find that parents trust doctors appearing even-handed in their judgements and medical opinions, for example, when they feel they give them encouragement without creating false hope. Meert et al. [44], in a study on the parent-doctor relationship with paediatric patients at risk of imminent death, indicated that some parents were convinced that doctors intentionally hid information to keep them optimistic and reduce their suffering, and this conviction negatively affected their degree of trust in professionals. Additionally, parents place more trust in doctors when they perceive their modesty, reflected, for example, in the fact that they admit in a matter-of-fact way their ignorance of some specific aspect of the disease or its treatment, if this occurs:

*It’s not that doctors don’t know how to diagnose it, it’s just, if they haven’t seen any case before … you know? Well obviously they won’t know what to do. But then, I’ve also come across, a bit, what you’d call medical pride, right? I mean you’ve got this doctor … yes, it’s true, I’ve seen that myself, you know? So, in my case … the truth is, to me, medical pride means nothing, I really don’t care …*

*We’re a pain! We’re really a pain because, I put myself in the doctor’s shoes, us families never stop bombarding them with questions. And so there are some, as with any human being, well, you don’t know how to answer. And if you’re a doctor, it’s like, “what do I tell them?”*

*Then you find doctors who say to you: “Oh, actually, I can’t”, or “Actually, look, call me in a while, because now, so and so … ”. And … and … I believe we’re human and, as humans, and as this kind of professional deals with so many diseases, it’s very difficult for them to be on top of everything. And you, you knock on the door and you’re there, and they’ve got no time to get ready. I mean, as much as they may want to, they don’t have the information to help you.*

Hsiao, Evan and Keltzer [29, 32] state that parents call for an understandable vocabulary, a direct style, clear explanations and complete information. They frequently mentioned their difficulties in understanding medical information, interpreting the treatment guidelines or knowing when and how to act in certain situations, and trust doctors when they perceive that the latter make an effort to be understandable. Doctors, as Dellve [45] notes, must have advance knowledge of parents’ competences to make sure that the level of complexity or detail of their explanations are suitable.

One interesting question is: who is more responsible for ensuring that the information has been fully understood. Is it the responsibility of parents to ask everything over and over again until they are sure they have understood it? Or is it the doctors who, as a part of their everyday routine, should include questions to ensure they have understood?

*The little they explain, we understand well. And what we don’t understand, we keep it in mind, or jot it down, and then look it up later on. In other words, I think that, yeah, we understand things. That’s why it’s hard to understand why they don’t do more explaining [when] we are able to understand. I guess they must sometimes think that we don’t have the ability to understand what they are going to explain to us. So, I don’t … perhaps they do it as a favour to us. I don’t know. I don’t think that’s much of a favour. I want to know everything. And I’ve also met other parents who’ve told me they don’t ask the doctor anything. They don’t ask because they won’t understand it and also because they’re scared of knowing so much. They prefer not to know.*

*“Do you understand?”. “Yes, yes, yes”. “And then, in a month’s time, we’re going to give him an endoscopy, or we’re going to do this or that”. “Do you understand?”. “Yes, yes, fine”. “Please explain it to me then”. And the family says: “What?” “So, you don’t understand, do you? Please explain it to me”. Well, that doesn’t happen. They don’t say: “So, family, explain what I’ve said back to me”. “Well, umm, err … ”. “So, you don’t understand”. In other words, here you realise … but the doctor doesn’t do that. The problem is that the family’s told the doctor, “I understand”. “OK, great, bye. Take care!”*

Generally for the parents, ‘bad’ doctors are those who display attitudes of arrogance and superiority, and who do not accept parents’ medical suggestions, regarding them as interference or challenges to their authority. Parents perceive an exaggerated ego and an inappropriate sense of ‘closed shop’, and all of this becomes a source of conflict. Dessy [46] notes in his study that, when communication between parents and doctors is characterised by conflict, it leads to stress, and if this continues over time, “relations between medical staff and families worsen” (p.39).

When proactive parents come up against this sort of doctor, clashes—if not open and serious confrontations—will sooner or later occur. A number of parents told of experiences demonstrating tension and clashing positions, i.e. situations in which both parties are aware of the breakdown in trust and, therefore, the fact that the communicative relationship is damaged.

We also encountered numerous allusions to the problem of the lack of coordination between professionals or different parts of the healthcare system, be this hospitals and their internal organisation or health centres. Parents often fail to understand how these ‘avoidable errors’ can occur, and are irritated by what they see as bureaucratic nonsense or the slovenliness with which some professionals deal with things, making them unnecessarily complicated in the parents’ view. In any case, they classify many of these problems of lack of coordination as being due to a ‘bad’ system, rather than any ‘bad’ professional in particular.

### Trusting those appearing in favour of parental proactivity

To establish fluid communications and a relationship based on deep mutual trust, parents need to be sure that their doctors do not conceal information and that they too are searching for answers. The research questions are obvious: What variables cause proactive behaviour in parents? Is it a question of personality? Are all parents equally proactive from the very start of the illness, or do they become more so over time? Or vice-versa? What might cause one situation to occur over another?

Parents refer to the irrefutable fact that they are the ones who know their children the most and the best. This is not only because they have given birth to them and have raised them, or even because they are the ones who spend more time with them. It’s because they have overall knowledge of the child that they have gained in the natural contexts of the latter’s development, compared with the one-dimensional knowledge possessed by the doctors and healthcare professionals caring for their child, which is gathered from observation in artificial contexts (for the child), such as a laboratory, a doctor’s office or a test room. Sometimes, parents feel that doctors are not willing to acknowledge their status as possessing inside information on the child, and this could entail a risk to the trust placed in the professional:

*Really, they know a lot about medicine; there are things that I’ll never be able to argue with, but my son is my son. And, regarding my son, I’m undoubtedly the one who knows the most. So, there, sometimes, it’s where there are times of … not of confrontation, not that but … well, a bit of a tussle, like, let’s see how we’re going to work this one out.*

Parents place more trust in professionals who respect and even encourage their behaviour as searchers for information, something already noted by Budych, Helms and Schultz [47] and by Leonard [48]. Sometimes, however, they are aware that this behaviour might create tensions:

*I mean, it doesn't bother them when you say, “I’ve read”, or “I’ve seen”. If they have to confirm it, they’ll do so. ( … ) The thing is that we parents have a defect, the fact that we go in there, like, almost knowing more than them, almost knowing more than them. Those that want to know, of course.*

*I’ve been on radio programmes ( … ) that had a section on rare diseases and they called someone every week. Ah, well, one week they called us. And I went to explain it. Well, can you believe the coincidence: my GP was at that very moment listening to the radio and recognised my voice? And the following week I had an appointment with him—it was a coincidence!—and he said to me, “Were you on the radio last week?”. And I said, “Yep”. [And the doctor said] “Well, I was in the car and I was listening to you. Hey, you gave a fantastic explanation of what’s going on with your son”.*

*The doctor’s never said anything to me. He’s never felt annoyed or anything. He’s always answered my questions. I don’t know if privately, inside, maybe he might have … that I made him appear … uhh. I’ve not had the feeling that it bothered him, you know? Never. From the very start, I’ve been very, very inquisitive. Really.*

Asked about cooperation with the doctor, one father stated that, “it depends on the doctor and also on the complexity of the case”, but that, in theory, “from our standpoint, maximum cooperation is the goal: making ourselves available for anything the doctors may require, in other words, always”. And he made it clear that this meant not getting ahead of oneself and perhaps suggesting “things that may annoy the doctor”. And he concluded, “above all, to convey to them our wholehearted desire to help and cooperate”. Resendez et al. [49] (2000) have already noted that meeting the needs of the family means making them active agents in the decisions that are being taken.

In much the same way, parents place more trust in doctors that display a positive attitude towards the association(s) to which they may belong and who approve of their involvement. As noted above, associations are a kind of antidote to the feeling of abandonment and loneliness experienced by many families with a child suffering from a rare disease. They provide not only affective support but also, depending upon the association’s own proactivity, very useful information and guidelines for families, who find in them resources to combat the lack of knowledge and uncertainty that cause them so much anxiety.As one parent put it, “the best medicine a doctor can prescribe is the association’s address”. Empowered parents approach other association members to provide and seek information, and they do so with the same strength with which they give and seek comfort. A doctor can distinguish between these two types of ‘assets’, but parents have no reason to do so, as they and only they know what it is they need most urgently at any given time:

*When there’s a situation of a rare disease, especially when there’s not much information, then the doctor should say “Look, the situation is so and so. Take the report, but there’s also this association. Go there, because … ”. And this type of rhetoric, it has the families asking you questions, because they don’t dare to ask the doctor.*

Parents find in associations support of all kinds, and don’t understand why there are some professionals who refuse to endorse the work of these groups and do not encourage parents to get involved in them:

*I think it must be made very clear that doctors need it, and it’s important for doctors to side with the family, back patients’ associations, and try to go when there’s a conference or a parent’s workshop to get a more in-depth handle on the situation.*

*What we see is that doctors are a bit reluctant … umm … to tell us certain things. I don’t know if it’s because, maybe, sometimes, there’s a bit of a lack of psychology or something. There are some doctors who don’t have a good idea of what associations are or what they are for.*

How can it be that there are doctors who do not approve of the associations’ work, who do not regard them as an ally but as something that interferes with their work?

*Because an informed family is always much better than one that isn’t informed at all, because the doctor will always have to give them information, information that he or she may not know how to give them. Or maybe it’s not that they don’t know, but that a family will not take in the information in the same way they would if it were given to them by a patients’ association.*

Any statements in this particular regard should be made with caution, as we do not have the viewpoint of the doctors, but the perception of parents in this regard is very interesting:

*Maybe because they think (wrongly, as far as my experience is concerned) that these associations may interfere or get in the way, that they may give information to those involved that isn’t completely right. When, in fact, if a patients’ association works properly, what it does, actually, is support research, ensure that those affected get information. ( … ) These associations are aimed [not only] at therapies for the patients themselves, but also at the families. It also depends, of course, on what each individual association is like. I think that doctors are a little reticent in this regard because of that. Because, in a way, they may be put under a bit of pressure, not from the individual patient, but from a group of families, which is obviously a lot more powerful than any single patient.*

A professional’s attitude toward an association to which the family belongs not only impacts upon the degree of trust parents have in the former. At times, it determines it in its entirety:

*So, the first thing he said to me was, “that association … be careful, it’s a cult”. And that’s what I was going to say before about egos. So what was going on? Well, Doctor ‘A’, who worked with the association, was a competitor of this other doctor. So, you know, he was leery. Obviously, with that kind of attitude, we had no interest whatsoever in a doctor with opinions like that. Right? So we changed doctor.*

### Trusting professionals whose door is always open

All the parents interviewed made mention, one way or another, of how useful it is to be able to contact the doctor in charge of their children’s case at any time. Professionals offering this availability are deserving of more trust that those who do not. Often, these references are followed by comments reflecting the parents’ appreciation of the difficulties inherent in a doctor’s work. This is why they are particularly grateful for the possibility of getting in touch with the professional whatever the day or time:

*Because I remember and have a great deal of affection for the doctor, you see? He’s like our father. I … I … well having his mobile, having his home contact details, being invited to his home … of course, I thought to myself, “If he did this with every family, it’d be chaos!” But obviously, it’s not commonplace. He does it with those he has affection for, too.*

This reciprocity in ‘affection’ points towards a mutual trust that, once truly in place, means that the availability of or the accessibility to the professional is a natural outcome of the relationship built up with the family:

*I’ve been in the waiting room waiting to have lunch … with him! And they are extraordinary doctors! True points of reference, who also give you their mobile. And you say to yourself, “How is this possible?” And they’re happy! Happy. They love what they do! And that’s really great, so cool, and really, really nice.*

*I believe you can tell a doctor who does check-ups via WhatsApp or other messenger these days: “Hey, I’ve got this and this going on”. And they’ll answer when they can. “Hey, up his level of … And when you come, I’ll ask for an appointment for you and when that’s arranged, you’ll come and see me”. Or, “Give him more and let me know if he gets better”. “Great. He doesn’t have to come to the appointment anymore”. I mean, all this is necessary.*

Although this same interviewee accepted the professional’s right to limit their accessibility to when it was ‘right’, and not at any time:

*Once I was in a debate. I was at a conference and mentioned the matter of the mobile and a doctor got really pissed off and said: “I don’t have to give my mobile number to anyone”. That’s right.*

Parents are aware that availability can lead to some taking advantage, meaning contacting the doctor excessively and unnecessarily often, when the situation really doesn’t call for it:

*What’s more, I remember I once called the doctor on a Sunday, [and she] was on the beach, reading a book. Lying on the beach, sunbathing. ( … ) I called her. She didn’t answer. I called her again a few minutes later, and she picked up, “What’s going on, María?”. Obviously, what’s going on, she knew something was up. Because I don’t call her [to tell her] that [my son] has got a bit of a temperature, I wouldn’t … no way! I call her when there’s a difficult situation and I don’t know what to do.*

*Why would a family bother you on a Sunday if it wasn’t something serious? What’s more, you’ll pick up if … but, in a way, you’re their fall-back doctor, their companion. No other doctor can understand the situation.*

Availability goes hand-in-hand with this affectionate and humane treatment we mentioned earlier. Parents trust doctors who treat them with warmth and empathy, and their perception is that it is only in this way that the professional is really engaged with their child’s case:

*You’ve got someone who’s a point of reference as a doctor and who’s the person that’s going to be able, from then on, to provide you with information, [with whom] your going to be closest, and you’re going to have more contact with the doctor. Even to the point that many of them give you their mobile number in case anything comes up. I mean, all this means that, even though it’s not compulsory, the doctor gets involved. In our case, our doctor was completely committed to us.*

Hsiao, Evan and Zelter [29, 32] conclude that both parents and children themselves prefer to communicate with doctors who are easy to get in touch with at any time, who quickly pick up the phone, or who rapidly reply to emails, not so much because they meet their unexpected requests for information, but because it gives them a sense of security and peace of mind.

## Discussion

The main goals of this study were the identification of the key elements upon which parents base their communicative relationship with the doctor, and the conceptualisation of a model that integrates said elements to provide an account of the communicative dynamics between the two parties. With regard to the former goal, our analysis identified 21 categories, grouped into five blocks, with the core category being ‘adjustment of mutual trust’.

Based on the results obtained, and with regard to the second of our two goals, we propose a theory that explains the communicative experience we have been talking about. Set out below are some of its key aspects, and we also give some pointers as to how, in our view, research should progress to complete it and provide it with a broader perspective.

Doctors with whom parents achieve the greatest levels of trust are characterised by their ability to observe the child, their sensitivity, their tact, their humility and their sincerity. They are doctors who, in these parents’ eyes, are committed and involved in personal matters, provide parents with companionship, go beyond strictly clinical matters, make themselves greatly available and show empathy by easily putting themselves in the parents’ shoes, as well as humility in acknowledging the limits of their knowledge and resources. Parents understand the difficulties of medical work and are eager to cooperate with doctors, feeling sure of their ability to help due to their possession of direct, overall and intuitive knowledge of the child that the professional lacks. If this cooperation occurs with the minimum degree of sensitivity, humane treatment and compliance with the principles of transparency and honesty in the exchange of information required by parents, a great degree of trust is generated, bolstering both parties so that they can better achieve their common goal: providing the child with the best possible care.

Parents’ trust in the doctor caring for their child is *eroded* or called into question to the extent that they fail to observe some of the characteristics that we have defined as ‘sources’ of trust, and clearly becomes *mistrust* if they are convinced of the opposite of each of them.

In the communicative encounter between parents and doctors, trust helps bridge the gap between the parties, whilst mistrust helps widen it. This ‘gap’ is a metaphor for asymmetries in the possession and handling of information and for differences in the feelings and emotions at play when they interact. Variations in trust levels result in these asymmetries reducing or accentuating.

Communication between parents with children suffering from rare diseases and the doctors caring for the latter takes place within a very complex context. This complexity arises from the existence of a doubly asymmetrical situation:

- Affective asymmetry. For their part, parents are always affected by an emotional burden (explicit or latent) in their encounters with doctors. For theirs, doctors, due to the nature of their job, always attempt (with a greater or lesser degree of success) to set aside any emotional burden that may negatively affect their work.
- Informational asymmetry. There is an unequal distribution of the amount and kind of information managed by each of the parties in the encounters between them.
  The degree to which each of these parties is aware of this double asymmetry varies, as does the way they attempt to even it out. No matter how strongly it impacts upon the parties’ awareness, the double asymmetry means they are constantly readjusting their mutual trust, which can go in two directions:

- Reduced asymmetry: when one (or both) of the parties develops a greater level of trust in the other, some of the asymmetries tend to decrease, such that one or both parties perceive a greater closeness or similarity in their positions.
- Accentuated asymmetry: when, as a result of the adjustment, the encounter between parties results in a lower level of trust on the part of one or both of them, one or more of the asymmetries are accentuated.

There is a need to identify other properties—and their associated dimensions—of the core ‘mutual trust’ concept-category.

In addition to the ‘sources’ of this trust, we believe that another interesting property is to be found in the ‘adjustment/readjustment processes’ and in the ‘consequences’ of an increase or decrease in trust.

However, to progress in this direction, there is a need for more (and more precise) data. And, obviously, when talking of mutual trust, we need to understand the viewpoint of the other party, i.e. the doctor’s. What would the sources of trust placed by the doctor in the patient’s family be? There is undoubtedly a great deal of research that needs to be done to complete an overall scorecard of the process. How do the parties readjust their mutual trust in their encounters? What factors play a part in the way the parties perceive asymmetries? What affects the direction the adjustment takes?

Our incipient theory suggests that there is one set of factors operating at a base level, such that the response to these questions points towards this set of factors. Firstly, we would have a set of factors associated with the parents, on whose basis we could distribute the latter’s variability along a spectrum. At one of the ends of this continuum would be the most proactive, most empowered parents, those who are more ‘voracious’ in the search for information, more committed (in terms of time, energy, etc.), more concerned, more ‘active’ in supporting their children, etc. This would be the end represented by the ‘sharks’, to borrow a term used by one of the mothers interviewed.

At the other end would be those parents who appear more passive, more resigned, more compliant with the doctor’s opinions: parents who, at the very end of the spectrum, would seem to settle for anything, and appear to simply sit back and passively observe what is happening to their children, the ‘happy flowers’, as the same mother put it. There is not enough data to be able to more accurately define the behavioural parameters we are using to describe parents at one specific point along the continuum, and the space between these two ends is host to a broad spectrum of mothers and fathers. Likewise, the positions are not fixed, as a wide variety of circumstances may cause shifts along this continuum, probably more frequently in the less to more proactive direction than the other way around.

Additionally, we would have a second set of factors associated with the doctor, which constitute another grouping upon whose basis we could distribute doctors’ variables along a different continuum. At one of the ends of this continuum would be those doctors we could call, using a provisional term, ‘open profile’ doctors. These are doctors who (in theory) inspire more confidence, are characterised by their greater emotional closeness to the family, and are better skilled at providing companionship for parents in living with their children’s illnesses, more transparent in handling information and readier to share this information and accept suggestions. These are doctors in whom families perceive a great humility, sincerity and honesty in all their encounters with them.

At the other end would be those doctors with a ‘closed profile’, characterised by their emotional distance from the family. They are also less skilled or just not interested in providing families with companionship in coping with their child’s disease, less transparent in handling information and less ready to share this information or accept suggestions. These are doctors in whom families perceive coldness, little humility, the concealment of information, a lack of professionalism, sometimes even malpractice, in their encounters with them.

In any case, we are aware of the current limitations of our study. The sample of parents interviewed reflects the profile of proactive parents. Agreeing to be interviewed means these parents are strongly committed to the treatment of their children’s disease, and that they are prepared to explain this experience and be recorded if they believe this might help increase awareness of the disease or uncover tools to help meet their needs. However, given the methodological framework we have employed (grounded theory), we believe that we are laying the foundations for further research and, in this sense, there is (up to a point) no need to control the characteristics of this initial sample. However, now that we have placed on record the fact that there is a profile of proactive or empowered parents (the ‘sharks’) and another of resigned or passive ones (the ‘happy flowers’), we need to find parents with this latter profile and convince them to talk of their experiences. Only in this way can we have a complete vision of the matter and aspire to present a solid theory on trust as the cornerstone for parent-doctor communication in the field of children suffering from rare diseases.

## Conclusions

In this study:

- We have identified key elements and communicative dynamics between parents of children with rare diseases and healthcare professionals.
- We have presented, in great detail, the sources of trust used by these parents in their communicative relationships with healthcare professionals.
- We have identified two additional properties of trust, namely *adjustment/readjustment processes* and *consequences*, which require more research to be defined in their different dimensions.

It is not the aim of this article to make concrete recommendations about the education and training of future health professionals or about how health services should be organised to facilitate closer and more fluid communication between families or patients and the professionals themselves. However, it is difficult not to be aware of the practical implications of our findings; even more so when parents’ trust in doctors is based on aspects that have to do with ethical values, namely empathy, transparency and respect, rather than with knowledge in itself.

This is especially relevant in the case of rare diseases, since very often it is not possible to achieve a cure and the health professionals’ primary concern is therefore the care of the patient and their family. This change of perspective (from cure to care) must be made explicit, taught and understood by doctors and health personnel as an essential part of their job.

## Additional files

Additional file 1: Interviews guidelines for data collection. (DOCX 19 kb)

Additional file 2: Blocks and categories built through the analysis. (DOCX 7 kb)

## Data Availability

The datasets used and/or analysed during the current study are available from the corresponding author upon reasonable request.
