# Supplementary material for: The experience of parents of children with rare diseases when communicating with healthcare professionals: towards an integrative theory of trust
Source: Orphanet J Rare Dis. 2019 Jun 28;14:159. doi: 10.1186/s13023-019-1134-1 (PMC6599337; doi:10.1186/s13023-019-1134-1)
Supplement: Supplementary file 1 — Interviews guidelines for data collection. (DOCX 19 kb) [file 13023_2019_1134_MOESM1_ESM.docx]

**INTERVIEWS “TOGETHER”**

1. INTRODUCTION
2. The interviewer introduces him or herself
3. Research objectives and interview objectives
4. Non-structured in-depth interview. Predicted maximum interview duration: 60 minutes
5. May I record the interview? Ask for permission to record the interview
6. Hand over a copy of the informed consent
7. INTERVIEW

1. Start with general questions. For example:

- 1. May I ask you first about your son/daughter?
  2. To get to know each other:
     1. What is his/her name?
     2. How old is he/she?
     3. How long did you have to wait for a diagnosis?
     4. How would you describe the treatment and your day-to-day life since you were given the diagnosis?
     5. How is he/she feeling at the moment?
     6. How do you feel as parents?

**Interviews:** We start from the fact that we will be talking about a rare disease and a minor child. Your role as parents is a long way from the role traditionally assigned to a patient or a patient’s parents. You seek out information and you are knowledgeable about your child’s disease. You play an active role.

- - How do you feel in this active role?
  - How do you think this active role influences your relationship with the doctor?
  - So, the sort of relationship you have with the doctor, which you have described to me, has it helped you with the treatment and in your day-to-day life or with other issues? Or it has been an inconvenience?

**Other questions**:

- Do you find the doctor’s explanations easy to understand?
- Do you find the medical reports easy to understand?
- Do the doctors give you all the medical information in writing?
  - Do you get any sort of follow-up or support by phone?
  - Do you get any complementary or support information by email?
- How would you like to receive medical information?
- APP: We are considering developing an app to help parents to understand medical information and facilitate communication and understanding. A pocket tool. If you could design the app, what would it ideally be like? Do you think that an app could help you with any other aspects related to the disease?
- Logbook: Keeping a record of treatment and other variables in your child’s day-to-day life and passing on this information to the doctor. What purpose do you think this information could serve?
