# Supplementary material for: The experience of parents of children with rare diseases when communicating with healthcare professionals: towards an integrative theory of trust
Source: Orphanet J Rare Dis. 2019 Jun 28;14:159. doi: 10.1186/s13023-019-1134-1 (PMC6599337; doi:10.1186/s13023-019-1134-1)
Supplement: Supplementary file 2 — Blocks and categories built through the analysis. (DOCX 7 kb) [file 13023_2019_1134_MOESM2_ESM.docx]

# ANNEX 2

## Blocks and Categories built through the analysis

## ***Block A, ‘Families’***

Category 1. AFFECTIVE IMPACT OF THE DISEASE ON PARENTS

Subcategory 1.1. ANYTHING FOR OUR CHILDREN

Subcategory 1.2. THE CHILD’S BEHAVIOUR

Subcategory 1.3. LUCK

Category 2. FAMILY DIVERSITY

Category 3. PROACTIVE/EMPOWERED PARENTS

Category 4. PASSIVE/RESIGNED PARENTS

Category 5. TRANSFORMATIONS IN FAMILY LIFE

## ***Block B ‘Doctors’***

Category 6. THE PROFILE OF GOOD DOCTORS

Category 7. THE PROFILE OF BAD DOCTORS

Subcategory 7.1. TENSION AND CONFRONTATION

Subcategory 7.2. LACK OF COORDINATION

## ***Block C ‘Families and information on their child’s disease’***

Category 8. THE SEARCH FOR INFORMATION

Category 9. ACCESS TO INFORMATION

Category 10. PROBLEMS IN UNDERSTANDING INFORMATION

Subcategory 10.1. THE FIGURE OF THE MEDIATOR

Subcategory 10.2. LOST PARENTS

Category 11. FAMILY MANAGEMENT OF THE INFORMATION

Category 12. THE USEFULNESS OF AN APP

## ***Block D ‘Parent-doctor communication’***

Category 13. UNDERSTANDING THE DIFFICULTIES OF MEDICAL WORK

Category 14. COLLABORATION WITH DOCTORS

Category 15. PARENTS’ INSIDE INFORMATION ON THEIR CHILD

Category 16. MORE HUMANISED MEDICAL CARE

Category 17. **ADJUSTMENT OF** **MUTUAL TRUST** (core category)

## ***Block E ‘Associations’***

Category 18. ASSOCIATIONS

Category 19. SHARING INFORMATION AND EXPERIENCES

Category 20. DOCTORS AND ASSOCIATION

Category 21. THE DISEASES’ MEDIA PROFILE
